# Distribution of Parasites Detected in Stool Samples of Patients in Le Dantec University Hospital of Dakar, Senegal, from 2011 to 2015

**DOI:** 10.1155/2017/8296313

**Published:** 2017-05-15

**Authors:** Khadim Diongue, Mouhamadou Ndiaye, Mame Cheikh Seck, Mamadou Alpha Diallo, Yaye Dié Ndiaye, Aïda Sadikh Badiane, Daouda Ndiaye

**Affiliations:** ^1^Service de Parasitologie et Mycologie, Faculté de Médicine, de Pharmacie et d'Odontologie, Université Cheikh Anta Diop, BP 16477, Dakar, Senegal; ^2^Laboratoire de Parasitologie et Mycologie, Centre Hospitalier Universitaire Aristide Le Dantec, BP 5005, Dakar, Senegal

## Abstract

To identify the parasites responsible for intestinal parasitic infections diagnosed at Le Dantec University Hospital of Dakar, distribution of parasites detected in stool samples of patients was studied. From 2011 to 2015, 2578 patients were included in the study. A direct examination and Ritchie technique were performed as parasite search techniques. In total, 408 samples were positive showing 440 intestinal parasites; this corresponds to prevalence of 15.8%. Parasites were detected in monoparasitism (85.7%) and multiparasitism (14.3%). The most common species found in monoparasitism were* Entamoeba coli* (38.9%),* E. histolytica/dispar* (12.7%),* Giardia intestinalis* (8%), and* Ascaris lumbricoides* (7.3%). The most common associations were* A. lumbricoides-Trichuris trichiura* (3.6%) and* E. coli-G. intestinalis* (2.7%). Nonhospitalized patients were significantly more affected with 65.4% compared to hospitalized counterparts; and also there were more men (50.7%) than women. With 67.4%, adults were the most affected age group, while the elderly were less affected with only 7% (*p* = 0.5). This study shows increasing prevalence of intestinal parasitic infections over the years. So health education should be promoted in addition to the already begun mass treatment program. This would help to limit or even halt the spread of these diseases.

## 1. Introduction

Intestinal parasitic infections are recognized as neglected tropical diseases [1]. They are a global health problem causing morbidity in 450 million people [2]. These infestations are particularly prevalent in disadvantaged communities, particularly in tropical and subtropical areas, because of their hot and humid climate and also because health conditions are often faulty and/or access to drinking water is more difficult [1, 3]. Their prevalence depends on not only the geographical location but also various socioeconomic factors such as climate, hygiene, and age [4].

The objective of this study is to identify the parasite species responsible for intestinal parasitic infections diagnosed at Le Dantec University Hospital of Dakar, Senegal.

## 2. Patients and Methods

We carried out a retrospective and descriptive study at the parasitology and mycology laboratory of Le Dantec University Hospital of Dakar. Between 2011 and 2015, all patients received in the laboratory were included in the study for parasitological examination of stools with symptoms suggestive of intestinal parasitic infections.

The main collection tool was the bench registries specially designed for parasitological examination of stools. These registries collected information on age, sex, hospitalized or nonhospitalized status of the patients, and the year and the results of the examinations. The age was defined in four categories: children (below 15 years), young adults (15–30 years), elder adults (31–60 years), and elderly (over 60 years).

Stool specimens were sent to the laboratory promptly after collection, in a plastic jar, for hospitalized patients; they were collected, in the laboratory itself, for the nonhospitalized patients. The stool was treated by two techniques:

- Direct examination
- Ritchie concentration technique

For statistical analysis, the data were saved with Microsoft Excel 2007 software and transferred to Epi Info 7 where they were processed. The significance level of statistical calculations was set at 5% (*p* value < 0.05).

The following formulas have made it possible to calculate the parasitic indices:

- Simple parasitic index (SPI), which corresponds to the prevalence here, is equal to the percentage of parasitized subjects relative to the total parasitological examinations of stools carried out.
- Corrected parasitic index (CPI) is equal to the ratio of the number of parasites recorded on the number of total examinations multiplied by 100.

## 3. Results

### 3.1. Characteristics of the Study Population

A total of 2578 patients were included in the study with a sex ratio of 1.1. Patients' age ranged from 11 days to 91 years with a mean age of 28.56 years. The distribution of patients by age group was as follows: children, 723 (28%); young adults, 768 (29.8%); elder adults, 907 (35.2%); and elderly, 180 (7%).

### 3.2. Parasitic Indices and Evolution of Prevalence according to Years of Study

Of the 2578 stool samples examined, 408 showed the presence of intestinal parasites in monoparasitism, biparasitism, or triparasitism, corresponding to a SPI or prevalence of 15.8%. Of these confirmed intestinal parasitic infections, 440 strains belonging to sixteen species of intestinal parasites, including seven protozoa and nine helminths, were counted as a CPI of 17%.

**Figure 1:**
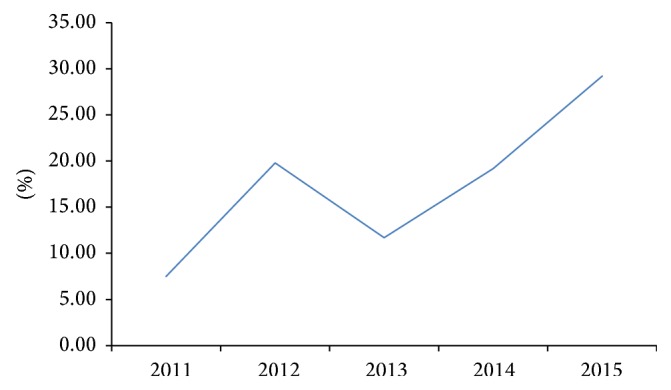
Evolution of the intestinal parasitic infections according to years.

The evolution of prevalence over the years showed a significantly increasing trend (*p* < 0.001) from 7.5% in 2011 to 29.2% in 2015. However, between 2012 and 2013, it decreased from 19.8 to 11.7% (Figure 1).

### 3.3. Distribution of Infestation according to Age, Sex, and Hospitalized or Nonhospitalized Status

These intestinal parasitic infections were significantly less (*p* < 0.001) in hospitalized patients (34.6%) than in nonhospitalized patients (65.4%). Regarding their distribution according to gender, a slight nonsignificant difference (*p* = 0.5) was observed with 207 male subjects (50.7%) compared to 201 female infected subjects (49.3%) (Table 1).

**Figure 2:**
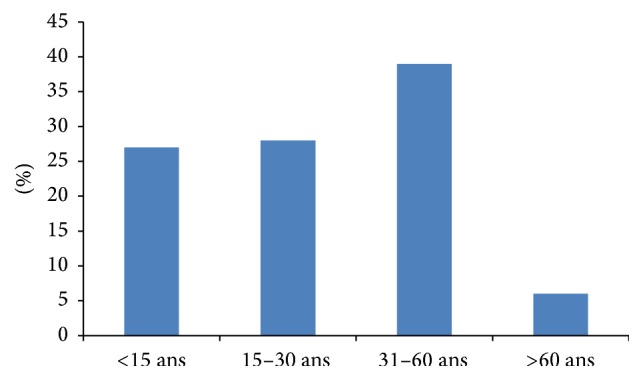
Intestinal parasitic infections' repartition according to age groups.

With 67.4% of confirmed intestinal parasitic infections, adults (15–60 years) were the most affected age group with more elder adults (39%) than young adults (28.4%), while the elderly were less affected with only 6.1% (Figure 2). However, this distribution according to age group was not significant (*p* = 0.96).

### 3.4. Distribution of Identified Species

Of these 440 identified intestinal parasites, 377 (85.7%) were identified in monoparasitism including 302 protozoa and 75 helminths. The most common protozoa were* Entamoeba coli*, 38.9% (171/440),* E. histolytica/dispar* (12.7%), and* Giardia intestinalis *(8%), while the most representative helminths were* Ascaris lumbricoides* (7.3%),* Trichuris trichiura* (5.5%), and* Taenia *sp. with 1.4% (Table 2(a)).

In biparasitism (13.6%), 60 parasites were identified with, first, associations between protozoa dominated by* E. coli-G. intestinalis* with 6 cases and* E. coli-E. histolytica/dispar* with 4 cases and then between helminths in which the only recovered association was that between* A. lumbricoides and T. trichiura* found 8 times; and finally for associations between protozoa and helminths, the most representative of which was that between* A. lumbricoides* and* E. coli* found 4 times (Table 2(b)).

Only one triparasitism case (0.7%) was observed with* A. lumbricoides-T. trichiura-E. histolytica/dispar* (Table 2(b)).

## 4. Discussion

Intestinal parasitic infections are a global health problem because of their morbid nature. They are due to different species of parasites varying with period and geographical region. It is in this context that this study was carried out within the laboratory of parasitology and mycology of Le Dantec University Hospital of Dakar during the period from January 2011 to December 2015 in order to identify the distribution of the responsible species. Overall prevalence of 15.8% was found.

This prevalence may be considered low compared to not only that found in another Dakar University Hospital laboratory where Sylla et al. found prevalence of 26.8% between 2006 and 2010 at the Fann hospital [5] but also that found in a study of slaughterhouse workers in Dakar with 49.56% even if the latter may be classified among subjects at risk [6].

Elsewhere, but still in West Africa, a study among schoolchildren in three regions of Mauritania found 33.4% prevalence of intestinal parasitic infections [7].

In the Maghreb, prevalence of 68.1% was found in rural areas among schoolchildren in Morocco in 2009 [8].

These values, especially the last one, are very high above ours. However, these differences could be put into perspective, given that these cross-sectional studies had been carried out in a population aged between 5 and 15 years, where hygiene conditions remained much more precarious, especially with promiscuity.

This same trend was also observed in the South American intertropical area, with prevalence of 70.7% which was found in Brazil in 2005 [9].

On the contrary, in Turkey, the prevalence found (3.7%) by a study carried out between 2012 and 2014 was four times lower than ours [10]. This low prevalence may be justified by the fact that intestinal parasitic infections are more frequent in developing countries (30 to 60%) than in developed ones (≤2%) [11]. However, taking into account the distribution of prevalence by years, we note that, in 2011, with 3.7%, our result was exactly equal to that found in Turkey.

We found intestinal parasitic infections higher in women (50.7%) than in men (49.3%) but without significant difference (*p* = 0.96). An opposite trend with similar proportions was found in Malaysia in a study on intestinal protozoa with 51% of men versus 49% of women [12]. This undoubtedly shows that gender does not necessarily influence infestation by intestinal parasites.

Regarding hospitalized or nonhospitalized status, intestinal parasitic infections were significantly more frequent in nonhospitalized patients with 65.4% than in hospitalized patients with 34.6%. This same observation was made studying the epidemiological aspects of intestinal parasitic infections diagnosed at the Fann hospital in Dakar [5]. This observation can be explained on one hand by the fact that very often patients considered nonhospitalized (outpatients) are, for the majority, hospitalized in other structures without a laboratory of parasitology. So they are actually hospitalized patients. On the other hand, the aim of the parasitological examination in outpatients, in general, is to confirm intestinal parasitic infection before treatment unlike in hospitalized patients (inpatients) in whom, very often, the parasitological examination of stools could aim to rule out the hypothesis of intestinal parasitic infection.

The distribution of infestation according to age group was not significant in our series with a higher frequency of infestation in adults with 67.4% and lower frequency in the elderly with 6.1%; between the two groups were the children with 26.5%.

This same trend was observed for intestinal parasitic infections diagnosed at the Fann University Hospital in Dakar between 2006 and 2010 [5]. On the other hand, this distribution of intestinal parasitic infections according to age is contrary to what has been reported in Morocco, where patients under the age of 18 years were the most infected with 80%, while patients over 18 years were the least affected with only 20% [13].

The species found in our studies remain with a few exceptions, the same found throughout the world, although the specific species' prevalence may vary over time and from one region to another [13].* E. coli* (38.9%),* E. histolytica/dispar* (12.7%),* G. intestinalis* (8%), and* A. lumbricoides* (7.3%) were the most found species in our series.

These same species have already been found among the predominant species by El Guamri et al. and Baba et al., respectively, in Morocco in 2009 and Mauritania in 2012 [7, 13]. However, the order of distribution could be different.

*E. coli* was the most frequent parasite found with 38.9%. This may be justified by the commensal nature of this amoeba considered to be little or not pathogenic [14].* E. histolytica* and* G. intestinalis*, both pathogenic, followed with 12.7% and 8%, respectively. They were found with similar proportions in Man in Côte d'Ivoire during a study of the prevalence of protozoa in students [14]. There, they were also found in association with a rate of 1.9% contrary to our study where they were not found together. The associations of protozoa which we found were dominated by* E. coli-G. intestinalis* (2.7%) and* E. coli-E. histolytica* (1.8%). Dhital et al. observed the latter association, with 1.7% in 2016 in Nepal [4].

Concerning helminths, a single association between helminths was found at 3.6% with* A. lumbricoides* associated with* T. trichiura* and especially associations between helminths and protozoa. The latter were also reported in Nepal in 2016 with 2 cases by Dhital et al. [4] who also found a triparasitism associating protozoa, while our study also found a triparasitism but with two helminths (*A. lumbricoides* and* T. Trichiura*) and a protozoan* (E. histolytica/dispar)*.

The associations found in our study showed very often the species considered as little or not pathogenic as* E. coli* or* T. intestinalis*, which confirms the opportunistic and frequent character of these protozoan species which, in the presence of favorable factors, can increase in number and determine digestive disorders.

## 5. Conclusion

Intestinal parasitic infections are found in Dakar (Senegal) with low prevalence compared to those of the subregion but they have an increasing trend. The parasites responsible are both protozoa and helminths but with predominance of the first. They are found in monoparasitism, biparasitism, or triparasitism. The protozoa species remain dominated by* E. coli* considered as little or not pathogenic species followed by* E. histolytica/dispar*, whereas the most frequent helminths were* A. lumbricoides* and* T. trichiura*. So health education needs to be promoted in addition to the mass treatment program that has already begun. This would help to limit or even halt the spread of these diseases, which remain a burden in developing countries.

## Figures and Tables

**Table 1:** Repartition of infestation according to gender and hospitalized or nonhospitalized status of the patients.

Variables	Total	%	*p value*
*Patients' status *			
Hospitalized	141	34,6	
Nonhospitalized	267	65,4	<0,001
*Gender*			
Male	207	50,7	
Female	201	40,3	0,5

**Table:** (a) Species distribution in monoparasitism

Species	Total	%
*Entamoeba coli*	171	38,9%
*Entamoeba histolytica/dispar*	56	12,7%
*Giardia intestinalis*	35	8%
*Ascaris lumbricoides*	32	7,3%
*Trichuris trichiura*	24	5,5%
*Blastocystis hominis*	18	4%
*Trichomonas intestinalis*	18	4%
*Taenia saginata/solium*	6	1,4%
*Strongyloides stercoralis*	4	0,9%
*Hymenolepis nana*	3	0,7%
*Schistosoma mansoni*	3	0,7%
*Isospora belli*	2	0,45%
*Endolimax nanus*	2	0,45%
*Ancylostoma*	1	0,2%
*Dicrocoelium dendriticum*	1	0,2%
*Enterobius vermicularis*	1	0,2%
*Total in monoparasitism*	*377*	*85,7 %*

**Table:** (b) Species distribution in multiparasitism

Species	Associated species	Number of isolations	Total of species	%
*Biparasitism*				*13,6 %*

*Between helminths*				

*Ascaris lumbricoides*	*Trichuris trichiura*	8	16	3,6%

*Between protozoa*				

*Blastocystis hominis*	*Entamoeba coli*	1	2	0,45%

*Blastocystis hominis*	*Trichomonas intestinalis*	1	2	0,45%
*Entamoeba coli*	*Trichomonas intestinalis*	1	2	0,45%
*Entamoeba histolytica/dispar*	*Entamoeba coli*	4	8	1,8%
*Entamoeba histolytica/dispar*	*Trichomonas intestinalis*	1	2	0,45%
*Giardia intestinalis*	*Trichomonas intestinalis*	1	2	0,45%
*Giardia intestinalis*	*Entamoeba coli*	6	12	2,7%

*Between protozoa and helminths*				

*Entamoeba coli*	*Ascaris lumbricoides*	4	8	1,8%

*Entamoeba coli*	*Strongyloides stercoralis*	1	2	0,45%

*Giardia intestinalis*	*Trichuris trichiura*	1	2	0,45%

*Giardia intestinalis*	*Strongyloides stercoralis*	1	2	0,45%

*Triparasitism*				

*Ascaris lumbricoides-Trichuris trichiura-Entamoeba histolytica*		1	3	*0,7 %*

*Total in multiparasitism*		*31*	*63*	*14,3 %*

## References

[1] Sahimin N., Lim Y. A. L., Ariffin F. (2016). Migrant workers in malaysia: current implications of sociodemographic and environmental characteristics in the transmission of intestinal parasitic infections. *PLoS Neglected Tropical Diseases*.

[2] Derso A., Nibret E., Munshea A. (2016). Prevalence of intestinal parasitic infections and associated risk factors among pregnant women attending antenatal care center at Felege Hiwot Referral Hospital, northwest Ethiopia. *BMC Infectious Diseases*.

[3] Nundu Sabiti S., Aloni M.-N., Linsuke S.-W.-L. (2014). Prévalence des géohelminthiases chez les enfants à Kinshasa. *Archives de Pediatrie*.

[4] Dhital S., Pant N. D., Neupane S. (2016). Prevalence of enteropathogens in children under 15 years of age with special reference to parasites in Kathmandu, Nepal; a cross sectional study. *SpringerPlus*.

[5] Sylla K., Tine R. C. K., Sow D. (2013). Aspects épidémiologiques des parasitoses intestinales diagnostiquées au laboratoire de parasitologie-mycologie du centre national hospitalier de fann. *Médecine d’Afrique Noire*.

[6] Ndiaye M., Badiane A., Seck M. C. (2015). Incidence des parasitoses intestinales chez les travailleurs d’abattoirs de Dakar. *Médecine d’Afrique Noire*.

[7] Baba O. A. S. C., Aminetou B. M., Ba O. (2012). Prevalence of intestinal parasites among school children in the Gorgol, Guidimagha and Brakna area (Mauritania). *Revue Francophone des Laboratoires*.

[8] Elqaj M., Belghyti D., Ahami A., Loutfi H., Elkharrim K., Taboz Y. (2009). Prévalence des parasitoses intestinales chez les écoliers en milieu rural Kenitra-Maroc. *World Journal of Biological Research*.

[9] Nascimento S. A., Moitinho M. R. (2005). Blastocystis hominis and other intestinal parasites in a community of Pitanga city, Paraná State, Brazil. *Revista do Instituto de Medicina Tropical de Sao Paulo*.

[10] Selek M. B., Bektöre B., Karagöz E., Baylan O., Özyurt M. (2016). Distribution of parasites detected in stool samples of patients admitted to our parasitology laboratory during a three-year period between 2012 and 2014. *Turkish Journal of Parasitology*.

[11] Abu-Madi M. A., Behnke J. M., Boughattas S., Al-Thani A., Doiphode S. H. (2016). A decade of intestinal protozoan epidemiology among settled immigrants in Qatar. *BMC Infectious Diseases*.

[12] Noor Azian M. Y., San Y. M., Gan C. C. (2007). Prevalence of intestinal protozoa in an aborigine community in Pahang, Malaysia. *Tropical Biomedicine*.

[13] El Guamri Y., Belghyti D., Achicha A. (2009). Enquête épidémiologique rétrospective sur les parasitoses intestinales au Centre hospitalier provincial El Idrissi (Kénitra, Maroc): bilan de 10 ans (1996–2005). *Annales de Biologie Clinique*.

[14] Ouattara M., Silué K. D., N’Guéssan A. N. (2008). Prévalences et polyparasitisme des protozoaires intestinaux et répartition spatiale d’*Entamoeba histolytica/Entamoeba dispar* et *Giardia intestinalis* chez des élèves en zone rurale de la région de Man en Côte-dIvoire. *Cahiers Santé*.

